# The SRS–Lenke–Aubin 3D classification of adolescent idiopathic scoliosis

**DOI:** 10.1007/s43390-025-01253-2

**Published:** 2025-12-16

**Authors:** Carl-Eric Aubin, Lawrence G. Lenke, Michael Vitale, Justin S. Smith, Virginie Lafage, Michelle C. Welborn, A. Noelle Larson, Takashi Kaito, Peter O. Newton, Jeffrey Mullin, Christiane Caouette, Brice Ilharreborde

**Affiliations:** 1 Department of Mechanical Engineering, Polytechnique Montréal, Downtown Station, P.O. Box 6079, Montreal, QC H3C 3A7 Canada; 2 Sainte-Justine University Hospital Center, 3175 Côte Sainte-Catherine Road, Montreal, QC H3T 1C5 Canada; 3 Department of Orthopedic Surgery, Columbia University Vagelos College of Physicians and Surgeons, NewYork-Presbyterian Och Spine Hospital, New York, NY USA; 4 Department of Orthopedic Surgery, Columbia University Vagelos College of Physicians and Surgeons, NewYork-Presbyterian Morgan Stanley Children’s Hospital, New York, NY USA; 5 Department of Neurosurgery, University of Virginia School of Medicine, Charlottesville, VA USA; 6 Department of Orthopaedic Surgery, Lenox Hill Hospital, Northwell Health, New York, NY USA; 7 Department of Orthopaedics and Rehabilitation, Oregon Health and Science University School of Medicine, Shriners Children’s Portland, Portland, OR USA; 8 Department of Orthopedic Surgery, Mayo Clinic, 200 First Street SW, Rochester, MN 55905 USA; 9 Division of Orthopaedic Surgery, Osaka University Graduate School of Medicine, Osaka Rosai Hospital, Osaka, Japan; 10 Department of Orthopedics, Rady Children’s Hospital, 3020 Children’s Way, San Diego, CA 92123 USA; 11 Department of Neurosurgery, Jacobs School of Medicine and Biomedical Sciences, University at Buffalo, The State University of New York, Buffalo, NY USA; 12 AP-HP Hôpital Universitaire Robert-Debré, 48 Boulevard Sérurier, 75019 Paris, France

**Keywords:** Lenke classification, AIS, Transverse plane deformity, 3D spinal classification, Axial vertebral rotation, Regional plane of deformation

## Abstract

**Purpose:**

Adolescent idiopathic scoliosis (AIS) is a complex three-dimensional (3D) spinal deformity, with transverse plane rotational components increasingly targeted by modern surgical techniques. Yet, clinical evaluation remains predominantly based on 2D radiographic parameters, including the widely used Lenke classification. In response, the 3D Classification Task Force of the Scoliosis Research Society (SRS) developed a comprehensive, clinically relevant classification system that remains intuitive, reproducible, and readily applicable by spine surgeons. This manuscript introduces the proposed SRS–Lenke–Aubin 3D classification and evaluates its ability to provide a structured and clinically meaningful 3D characterization of spinal deformities in AIS.

**Methods:**

To maintain continuity with standard clinical practices, the system builds upon the established Lenke classification and introduces two complementary 3D descriptors: the orientation of the regional plane of deformation (ORPD) and the apical vertebral rotation (AVR). These indices capture transverse plane deformities at the regional and local levels, respectively. Each was independently assessed for the proximal thoracic (PT), main thoracic (MT), and thoracolumbar/lumbar (TL/L) regions using calibrated 3D reconstructions from biplanar radiographs. A population-representative cohort of 285 AIS surgical cases was used to evaluate the system. ORPD and AVR values were translated into categorical modifiers using predefined clinical thresholds.

**Results:**

The new SRS–Lenke–Aubin 3D AIS classification adds two transverse plane modifiers per spinal region—the ORPD and AVR—yielding a modular 3-tiered, 4-modifier system: Curve type (1–6), Lumbar spine modifier (A, B, C), Thoracic sagittal profile modifier (−, N, +), Transverse plane regional modifier (ORPD: 1–3) and Transverse plane local modifier (AVR: s, m, l). A broad range of ORPD × AVR combinations was observed across the cohort, reflecting the system’s ability to capture transverse plane heterogeneity. Notably, similar coronal curve types often exhibited divergent transverse morphologies, underscoring the added value of these 3D descriptors in identifying clinically relevant variation.

**Conclusions:**

The SRS–Lenke–Aubin 3D classification enriches the existing Lenke framework by incorporating practical transverse plane descriptors compatible with standard imaging workflows. This system offers a clinically meaningful step toward more complete 3D characterization of AIS, with potential applications in improving surgical planning, assessing outcomes, and supporting future integration with automated 3D tools.

## Introduction

Adolescent idiopathic scoliosis (AIS) is a complex three-dimensional (3D) spinal deformity, primarily characterized by structural curves in the coronal plane, rotational deformities in the transverse plane, and alterations in sagittal spinal curvature. In severe cases, surgical treatment by spinal instrumentation and fusion is warranted. Traditionally, clinical evaluation and classification of AIS is performed on two-dimensional (2D) radiographic measurements, focusing mainly on coronal and sagittal deviations. These 2D descriptors form the basis of widely used classification systems, including the Lenke classification [1, 2], which identifies structural curves and incorporates two key modifiers: the lumbar spine modifier, reflecting coronal translational alignment of the lumbar apex, and the thoracic sagittal profile modifier, describing thoracic kyphosis (T5–T12). The Lenke classification has played a central role in standardizing AIS evaluation and guiding surgical planning, becoming the most frequently referenced system in the field of spinal deformity (e.g., [1], cited over 2412 times according to Google Scholar as of September 2025). By objectifying curve types and modifiers, the Lenke classification has become a common language with an accurate and reproducible basis for posterior spinal instrumentation and fusion strategies [3].

However, the 3D nature of AIS extends well beyond coronal and sagittal curvatures, encompassing concomitant vertebral rotation and spinal torsion in the transverse plane [4–7]. These transverse plane components may play a critical role in the clinical manifestation and progression of AIS, with direct implications for biomechanical function, cosmetic appearance, and the effectiveness of surgical correction. Yet, the current 2D modular Lenke classification system does not explicitly incorporate these aspects, limiting its capacity to fully capture 3D complexity of AIS. Some patients have a lot of rotation, whereas other have minimal rotation, which is not well-captured in the Lenke classification. A better language to understand the axial plane may aid in scientific communication, surgical planning, description of correction strategies and ultimately improved surgical results.

Advances in imaging technologies and computational 3D reconstruction algorithms have markedly improved the ability to assess spinal deformities in 3D, offering insights beyond what conventional 2D radiographs can reveal [6, 8, 9]. The advent of low-dose biplanar imaging systems has made 3D analysis more accessible [10, 11], marking a major step forward. However, their clinical use remains limited due to proprietary access, high costs, limited availability outside research settings, and adoption primarily confined to specialized centers. As a result, 3D evaluation remains largely absent from routine clinical workflows. In parallel to the development of low-dose biplanar imaging systems, advances in research have led to the development of validated self-calibrated reconstruction algorithms that have demonstrated the feasibility of generating 3D spinal models from standard radiographs acquired on any existing radiographic system, independent of imaging platform [6, 12–14] thereby increasing the potential for broader clinical adoption.

Parallel to these technological developments, several research groups have proposed 3D classification systems for AIS. Notably, Stokes et al. [15], under the auspices of the original SRS 3D Committee, used cluster analysis of 3D spinal shape, emphasizing the rotation of the plane of maximum curvature as a central parameter. This work underscored the relevance of incorporating multidimensional spinal shape descriptors into classification schemes and anticipated the need for segment-level 3D metrics that could complement existing 2D-based frameworks. Sangole et al. [16] further advanced this work by integrating vertebral axial rotation and 3D curvature orientation using unsupervised clustering. This led to a proposed 3D classification of thoracic scoliotic curves and the development of the da Vinci representation—a top-view schematic of the spine designed to summarize curve morphology across all three anatomical planes [6, 16]. In the same spirit, Illés et al. [9, 17, 18] highlighted the relevance of the axial plane by proposing a method for quantifying and visualizing vertebral rotation, reinforcing the value of axial metrics in routine scoliosis assessment. The central hip vertical axis (CHVA) was proposed as a standardized reference axis for 3D curve analysis [19]. Additional studies explored apical sagittal variability within Lenke 1 curves, highlighting the limitations of 2D-based classifications [20], as well as geometric torsion to distinguish subtypes [21, 22]. Shen et al. [23] proposed an integrative 3D classification that combines geometric and clinical parameters. Other contributions include classification models based on fuzzy clustering [24, 25], multivariate analysis [26], torsion-based descriptors [27, 28], and data-driven cluster analyses aimed at predicting surgical outcomes [29, 30].

Despite these important contributions, most advanced 3D technologies and proposed 3D classification systems have not transitioned into routine clinical use. Barriers such as limited accessibility, methodological complexity, and lack of clinical interpretability have hindered their integration into real-world workflows—particularly in surgical decision-making contexts [7, 31]. These challenges have underscored the need for a practical, standardized, and clinically meaningful 3D classification system that can be seamlessly integrated into real-world radiographic workflows—regardless of the imaging platform—and that supports informed surgical decision-making.

In response, the current SRS 3D Classification Task Force, convened by the Scoliosis Research Society, builds on the foundational work of the original SRS 3D Committee [6, 15]. SRS leadership tasked the committee to develop a comprehensible, reproducible, and clinically meaningful 3D classification system for AIS.

This manuscript presents the SRS–Lenke–Aubin 3D classification system, targeted for integration into routine clinical workflows, and future automation. It outlines the system’s potential to enhance 3D understanding, inform surgical planning, and support clinical communication. It also evaluates the classification’s clinical relevance and its ability to intuitively characterize 3D spinal deformities in AIS.

## Methods

The foundational premise of the proposed 3D classification system was to build upon the existing Lenke 2D classification [1, 2], which is already well-established, widely adopted, and routinely used in clinical practice by spine surgeons. The approach was to extend this familiar system by incorporating key descriptors that objectively capture transverse plane deformities, which are absent from the original 2D scheme. This design choice aims to facilitate clinical adoption, reduce conceptual barriers, and ensure broad applicability across healthcare settings, including those with limited access to advanced 3D imaging technologies. The guiding principles were simplicity, clinical relevance, and interoperability—acknowledging both conceptual and operational challenges related to integrating 3D descriptors into routine workflows. In response to this need, the SRS 3D Task Force developed the SRS–Lenke–Aubin 3D AIS classification as a natural evolution of the Lenke system.

Crucially, the system was intentionally designed for compatibility with a wide range of radiological platforms. This was made possible using a self-calibration method that does not require proprietary equipment. Anatomical data for 3D reconstruction were derived from calibrated coronal and sagittal radiographs, in DICOM or JPEG format. Key anatomical landmarks, including the vertebral body corners, endplate centroids, pedicle centroids, and center of the femoral heads, were identified either manually or through AI-assisted detection. The validated self-calibration algorithm [12–14, 32] was then used to reconstruct the 3D coordinates of these landmarks, with reported accuracy below 1.5 mm. The algorithm estimates projection geometry without requiring external calibration, facilitating ease of clinical use across diverse imaging systems. Spatial scaling was completed using a radiographic object, and a minor 3D model reorientation (generally < 3°) was computationally applied to ensure alignment of the femoral heads within the coronal plane. This step provides a consistent, patient-based reference frame that is independent of the radiographic acquisition position, which may slightly vary between views. A spinal reference system was then established using the central hip vertical axis (CHVA) [19] as the vertical axis, along with orthonormal axes defining the coronal, sagittal, and transverse anatomical planes. This system enabled the derivation of angular and positional descriptors of the deformity within each anatomical plane.

A key methodological choice was to restrict the number of descriptors to a concise yet clinically relevant set—sufficient to capture the essence of 3D deformity while remaining intuitive, communicable, and consistent with the logic of the original Lenke system. The complexity of 3D deformities was distilled into two core descriptors: one characterizing the regional shape of the scoliotic curves (analogous to the Cobb angle in the coronal plane), and another capturing vertebral rotation (or torsion) at the local level, similar to what is observed in axial CT imaging along the axis of symmetry of a vertebral body.

### Regional deformity characterization: orientation of regional planes of deformation (ORPD)

**Fig. 1:**
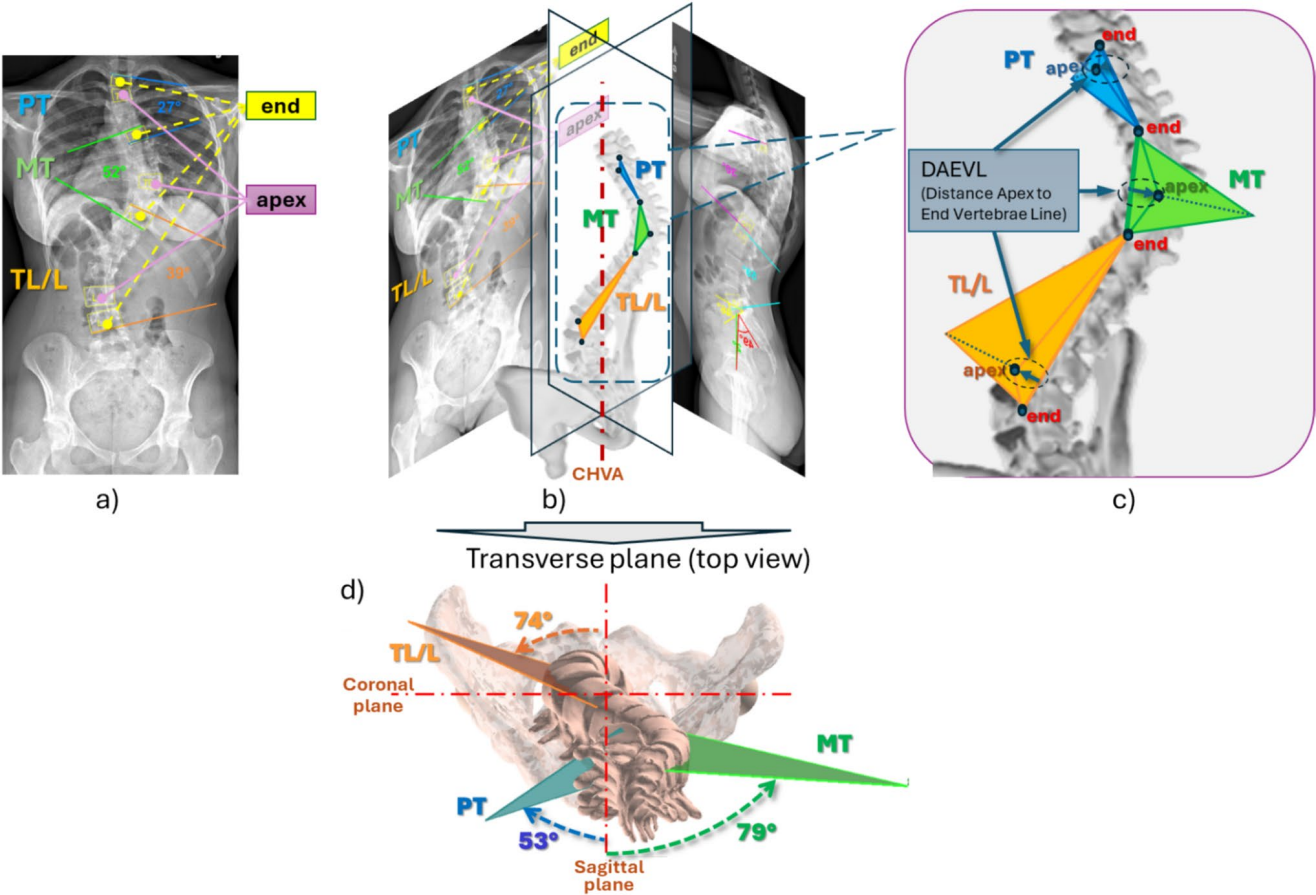
**a** Identification of end and apical vertebrae for the proximal thoracic (PT), main thoracic (MT), and thoracolumbar/lumbar (TL/L) segments, following the Lenke classification method. **b** Extraction of the 3D coordinates of the centroids of the end vertebrae and the apex for each segment, followed by construction of regional triangles (RPD) connecting these three points. **c** Magnified view of the RPDs, showing the proportional extension of each triangle toward the apex for enhanced visualization. For clarity, the apex arm is displayed at approximately three times the DAEVL in total. The distance between apex and end-vertebrae line (DAEVL) is also depicted. **d** Projection of the 3D spine reconstruction and RPDs onto the transverse plane (top view), with the horizontal axis aligned to the patient’s coronal plane and the vertical axis to the sagittal plane. This view illustrates the orientation of each regional plane of deformity (ORPD) relative to the sagittal plane and defines the conventions for positive angles. By convention, PT, MT, and TL/L segments are shown in blue, green, and orange, respectively

For each spinal region—proximal thoracic (PT), main thoracic (MT), and thoracolumbar/lumbar (TL/L)—the end and apical vertebrae are identified on coronal radiographs according to the Lenke classification (Fig. 1a). The corresponding 3D centroids of these vertebrae are extracted from the reconstructed model and used to define a triangular regional plane of deformation (RPD) for each spinal region. Each triangular RPD represents a spatially oriented plane that captures the position of the apex relative to the 3D line connecting the end vertebrae.

A key metric extracted from the RPD, the 3D distance between apex and end-vertebrae line (DAEVL), is defined as the perpendicular distance from the apical vertebra to the line joining the end vertebrae. This provides a spatial measure of regional curve convexity and apical deviation (Fig. 1c). The RPD visually integrates both scoliotic lateral displacement and anterior–posterior excursion of the apex due to sagittal curvature, offering a comprehensive 3D representation of regional spinal deformity.

Each RPD is then projected onto the transverse plane, perpendicular to the CHVA (Fig. 1d). In this top-view projection, the vertical axis corresponds to the sagittal plane and the horizontal axis to the coronal plane. The orientation of the projected RPD is assessed by measuring the angle between the RPD and the sagittal plane, defining the orientation of the regional plane of deformation (ORPD). In a normal spine, regional planes tend to align with the sagittal plane. In scoliosis, however, lateral curvature and sagittal profile changes cause deviation of these planes.

An ORPD is calculated for each of the PT, MT, and TL/L segments. For PT and MT—typically kyphotic—the posterior convexity causes the RPD to project posteriorly, behind the coronal plane. In contrast, the TL/L segment, generally lordotic and posteriorly concave, projects anterior to the coronal plane (Fig. 1d). Angular orientation is defined as positive from the sagittal plane: counterclockwise for MT (right-sided curve from the AP axis) and clockwise for PT (left-sided curve). Reversed curve directions are assigned negative values. For the TL/L segment, which is usually left-sided and lordotic, ORPD is also defined positively as counterclockwise from the sagittal plane.

For visualization, the RPD triangle is proportionally extended within its spatial plane toward the apical vertebra by a distance equal to twice the DAEVL, constrained between a minimum of 25 mm and a maximum of 125 mm (Fig. 1c). For clarity, this produces a displayed triangle whose apex arm measures approximately three times the DAEVL in total.

### Local deformity characterization: apical vertebral rotation (AVR)

At the individual vertebral level, axial rotation is defined as the angle between the local posterior-anterior axis of a vertebra and the patient sagittal plane. Because landmark identification can be affected by radiographic quality, acquisition variability, and anatomical overlap, the 2D and 3D measurement and 3D reconstruction algorithms incorporate robust estimation and error-compensation strategies to preserve accuracy. These strategies prioritize landmarks that remain consistently visible across views and apply geometric fitting methods based on multiple anatomical reference points. Together, these techniques improve the reliability of rotation measurements, minimize errors due to ambiguous landmarks, and ensure consistent vertebral orientation.

**Fig. 2:**
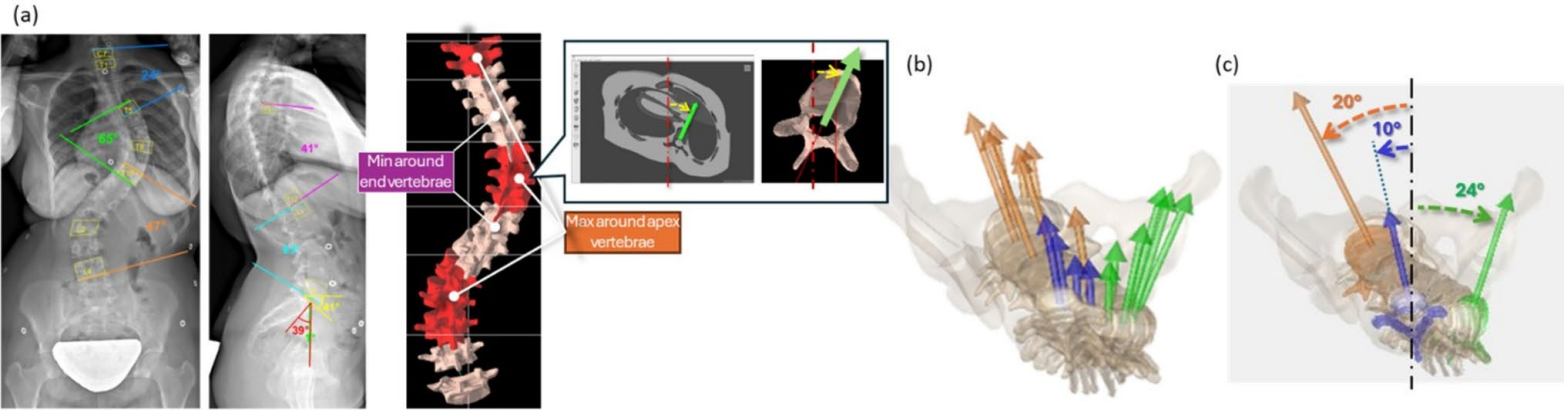
**a** Analysis of vertebral axial rotation along the spine, showing that maximal rotation typically occurs near the apex. A CT scan inset illustrates the concept of vertebral rotation relative to the sagittal plane. **b** The top-view projection depicts the axial orientation of each vertebra as an arrow, with its direction indicating the rotational angle relative to the sagittal plane and its length proportional to the rotation magnitude. **c** Top-view projection of apical vertebral rotation (AVR) for PT (in blue), MT (in green), and TL/L (in orange) segments, using the same representation. Angular values are indicated next to each arrow

Given that the greatest rotation usually occurs near the curve apex [33]—and to keep the classification framework simple—only the apical vertebral rotation (AVR) is retained for each of the PT, MT, and TL/L segments (Fig. 2). AVR is visually represented as an arrow whose direction indicates the orientation of the apical vertebral plane relative to the sagittal plane, and whose length is proportional to the magnitude of the rotation angle. This graphic representation is inspired by the schematic top-view vertebral orientation proposed by Labelle et al. [6] and the vertebral vector concept introduced by Illés et al. [17], with the distinction that, in the present system, arrow length reflects the magnitude of apical vertebral rotation rather than a normalized value. Angular values are displayed alongside each arrow. In typical curve patterns, AVR is clockwise in the MT segment and counterclockwise in the PT and TL/L segments (when viewed from above), reversed directions are denoted by negative values, in accordance with convention.

### Transverse plane modifiers and determination of subcategory thresholds

Building on the two descriptors (ORPD and AVR) introduced above, two additional transverse plane modifiers were integrated into the original Lenke classification framework: (i) a regional modifier based on the ORPD, and (ii) a local modifier based on the AVR. The ORPD modifier characterizes the spatial orientation of the spinal curve in the PT, MT, and TL/L regions, while the AVR modifier locally quantifies vertebral rotation at the apex of each curve. To preserve the intuitive and pragmatic structure of the original Lenke classification, the distribution of each index was analyzed and categorized into three clinically meaningful subgroups. This 3-tier schema was designed to ensure that the modifiers remain straightforward, interpretable, and useful in everyday surgical planning.

#### Regional Transverse Plane modifier (ORPD)

**Fig. 3:**
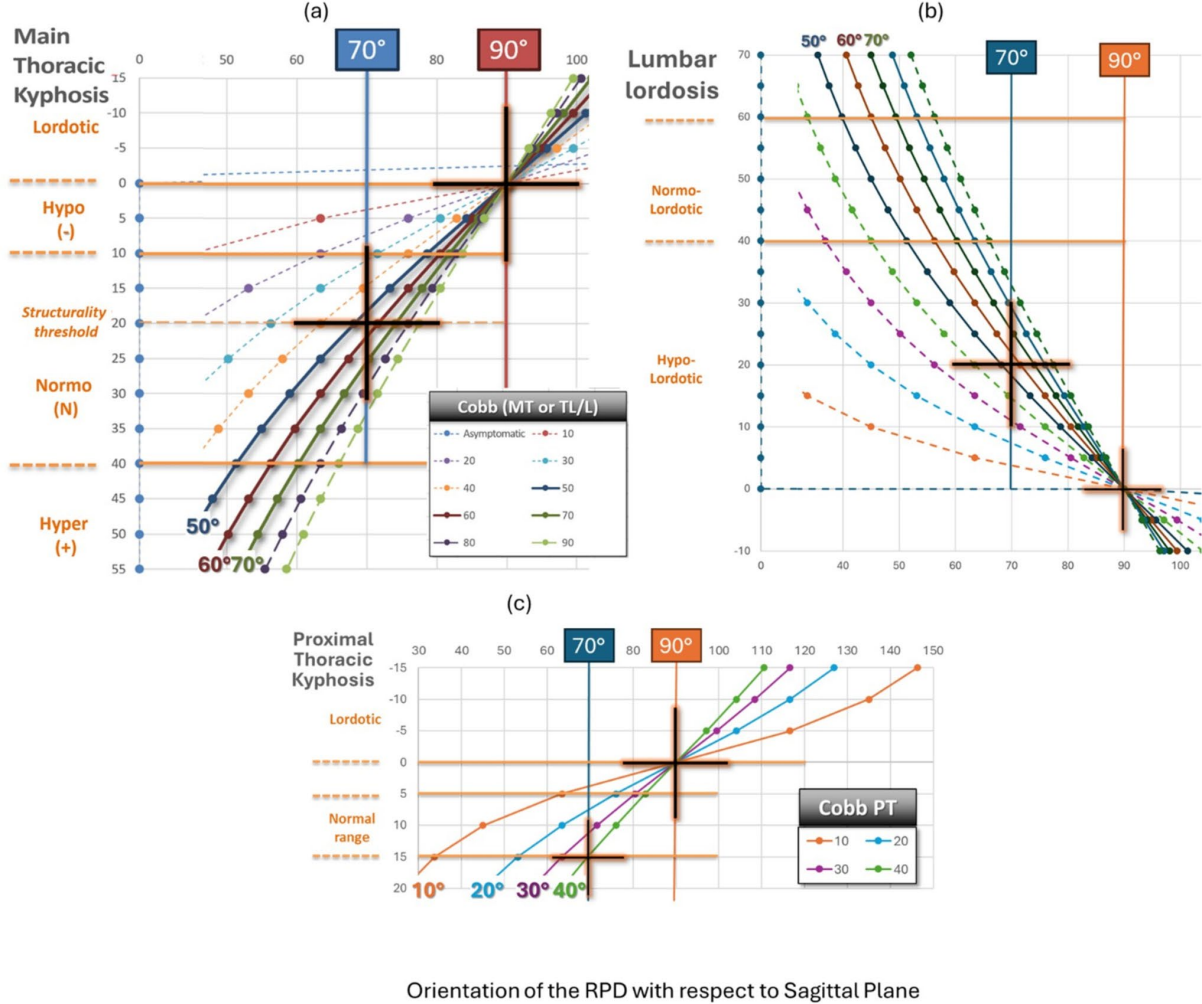
Theoretical relationships between sagittal and coronal curvatures and ORPD: **a** Relationship between thoracic kyphosis (TK) and MT Cobb angle, with illustrative curves ranging from asymptomatic spine (0° Cobb) to severe deformities (up to 90° Cobb), and the corresponding ORPD in the thoracic region; **b** relationship between lumbar lordosis (LL) and TL/L Cobb angle over the same Cobb angle spectrum, and the associated ORPD in the lumbar region; **c** Relationship between proximal thoracic kyphosis and PT Cobb angle over plausible Cobb angles spectrum, and the associated ORPD in the proximal thoracic region. The horizontal axis represents the ORPD with respect to the sagittal plane

**Fig. 4:**
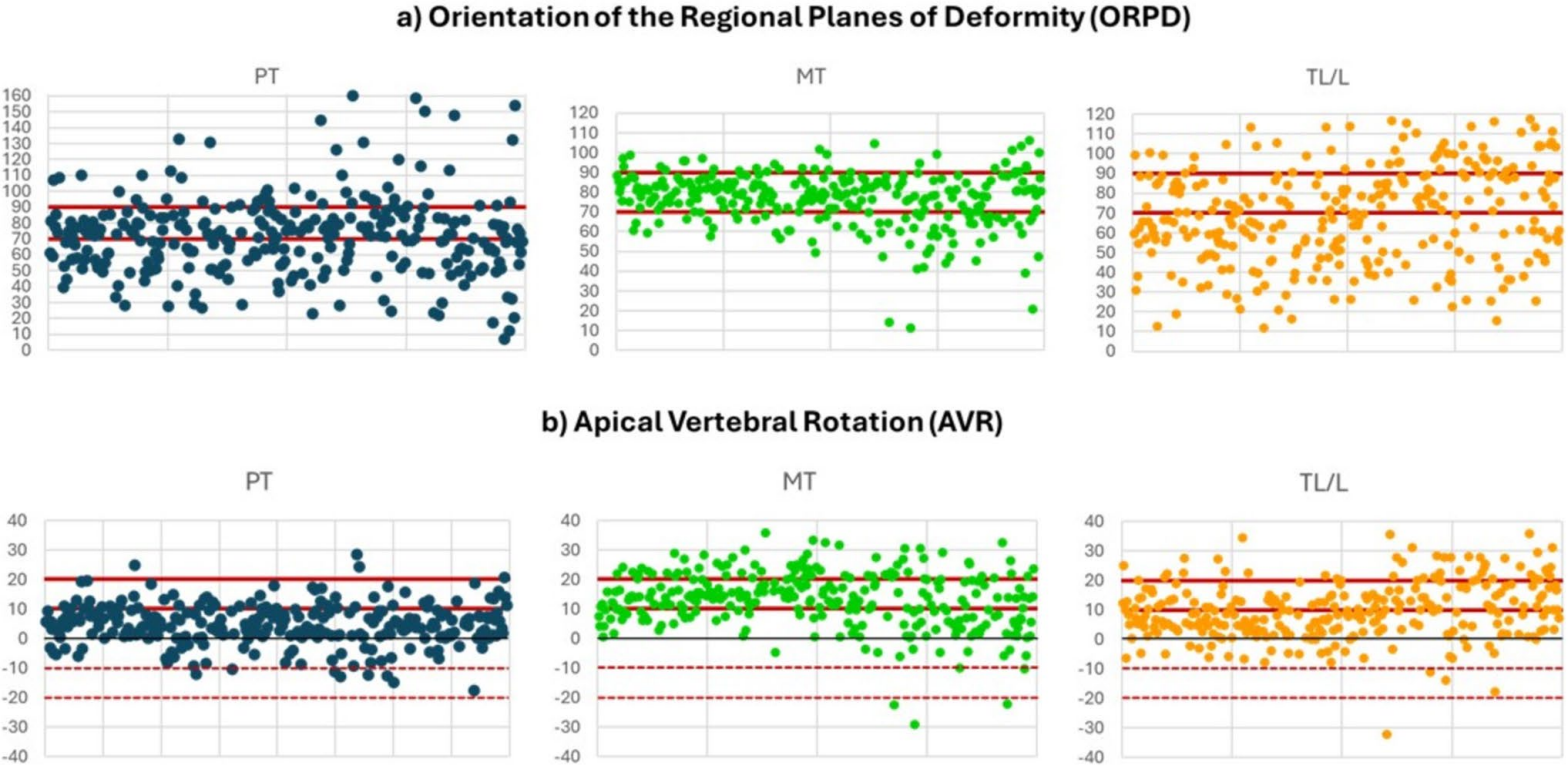
Distribution of transverse plane indices across PT, MT, and TL/L segments for 285 patients. Each dot represents an individual patient: **a** ORPD; **b** AVR

Thresholds for the regional transverse plane modifier were defined through a combination of a theoretical analysis of ORPD in relation to increasing Cobb angle and sagittal alignment (e.g., thoracic kyphosis and lumbar lordosis calculated over the same end vertebrae as identified in the coronal plane for the PT, MT and TL/L segment for consistency with the Lenke classification) (Fig. 3), supported by distribution data (Fig. 4a) and representative case studies. This led to the establishment of a three-tiered categorization system:

- Subclass 1 (< 70°): In MT segments with a coronal magnitude > 50°–55°, an ORPD < 70° typically corresponds to thoracic kyphosis > 20°, indicative of a structurally maintained kyphotic profile. Similarly, in TL/L segments, an ORPD < 70° is commonly associated with preserved lumbar lordosis (> 40°), suggesting a sagittal alignment consistent with physiological curvature and functional spinal balance.
- Subclass 2 (70°–90°): Intermediate ORPD values represent a transition zone, often associated with reduced thoracic kyphosis or lumbar lordosis in cases with surgically significant coronal curvature. In cases where coronal curve magnitude exceeds 50°–55°, an ORPD > 70° is typically observed when segmental thoracic kyphosis is < 20° or lumbar lordosis < 20°–30°, indicating increased axial deviation and sagittal flattening of the curve.
- Subclass 3 (> 90°): ORPD values exceeding 90° for MT curves, indicate that the apex lies anterior to the line connecting the end vertebrae (posterior for TL/L), reflecting a sagittal curvature reversal within the region they represent and a more pronounced transverse plane deformity. This configuration generally implies greater structural changes and may influence surgical realignment strategy.

While normative values for MT and TLL alignment in the sagittal plane are well established, reference data for PT kyphosis are more limited. Previous studies suggest a typical range of approximately 5°–15° in AIS and asymptomatic controls, with an average value centered around 10° (e.g., [34]. These values, however, vary with age, posture, and the specific spinal levels included, and are not consistently reported across sources. Given these limitations and for consistency, the same ORPD thresholds (70°, 90°) were applied to the PT segment, following a similar rationale—where an ORPD below 70° is indicative of a smaller Cobb angle or a greater proximal thoracic kyphosis. However, if the PT curvature is minimal or nearly straight (i.e., Cobb and sagittal angle ≤ 5°), or involves fewer than three vertebrae, the modifier is not reported and is denoted as “*”.

#### Local axial rotation modifier (AVR)

Thresholds for the local axial rotation (AVR) modifier were defined based on AVR distribution analyses (Fig. 4b) and clinical case review, resulting in a common three-tiered system applicable across the three spinal segments (PT, MT, TL/L). These thresholds were intended to stratify the severity of apical rotation into clinically meaningful categories, acknowledging both the increasing recognition of axial rotation in surgical planning—particularly for derotation maneuvers or fusion level selection—and the known variability in measuring this parameter. The classification was designed to balance interpretability and robustness, aligning with the Lenke system’s emphasis on simplicity while enhancing its 3D descriptive capacity:

- Subclass “s” (slight, < 10°): Reflects minimal rotation in the transverse plane. The vertebra remains closely aligned with the sagittal plane, often resembling non-structural or asymptomatic rotational deformities without notable mechanical impact.
- Subclass “m” (moderate, 10°–20°): Represents a moderate degree of apical vertebral rotation that may influence curve rigidity and factor into surgical decision-making.
- Subclass “l” (large, > 20°): Represents a marked torsional deformity at the apex, typically associated with a structural rotational component that may necessitate targeted derotational correction strategies.

### Integration of transverse plane modifiers into the extended Lenke classification

Building on the two newly introduced transverse plane descriptors—ORPD and AVR—and integrating them with the classic Lenke curve type, lumbar spine modifier, and thoracic sagittal profile modifier, the SRS–Lenke–Aubin 3D classification offers an expanded yet intuitive framework for characterizing AIS. This results in a comprehensive, modular, 3-tiered, four-modifier system, designed to capture the multidimensional nature of AIS while maintaining a clinically intuitive format:

$${\text{Curve Type }}\left( {{1} - {6}} \right) \, + {\text{ Lumbar Spine Modifier }}\left( {{\mathrm{A}},{\mathrm{B}},{\mathrm{C}}} \right) \, + {\text{ Thoracic Sagittal Profile Modifier }}\left( { - ,{\text{ N}}, \, + } \right)$$

$$+ {\text{ Transverse Plane Modifiers for PT}},{\text{ MT}},{\text{ TL}}/{\mathrm{L}}:{\text{ ORPD }}\left( {{1},{ 2},{ 3}, \, *} \right){\text{ and AVR }}\left( {{\mathrm{s}},{\text{ m}},{\text{ l}}} \right)$$

**Fig. 5:**
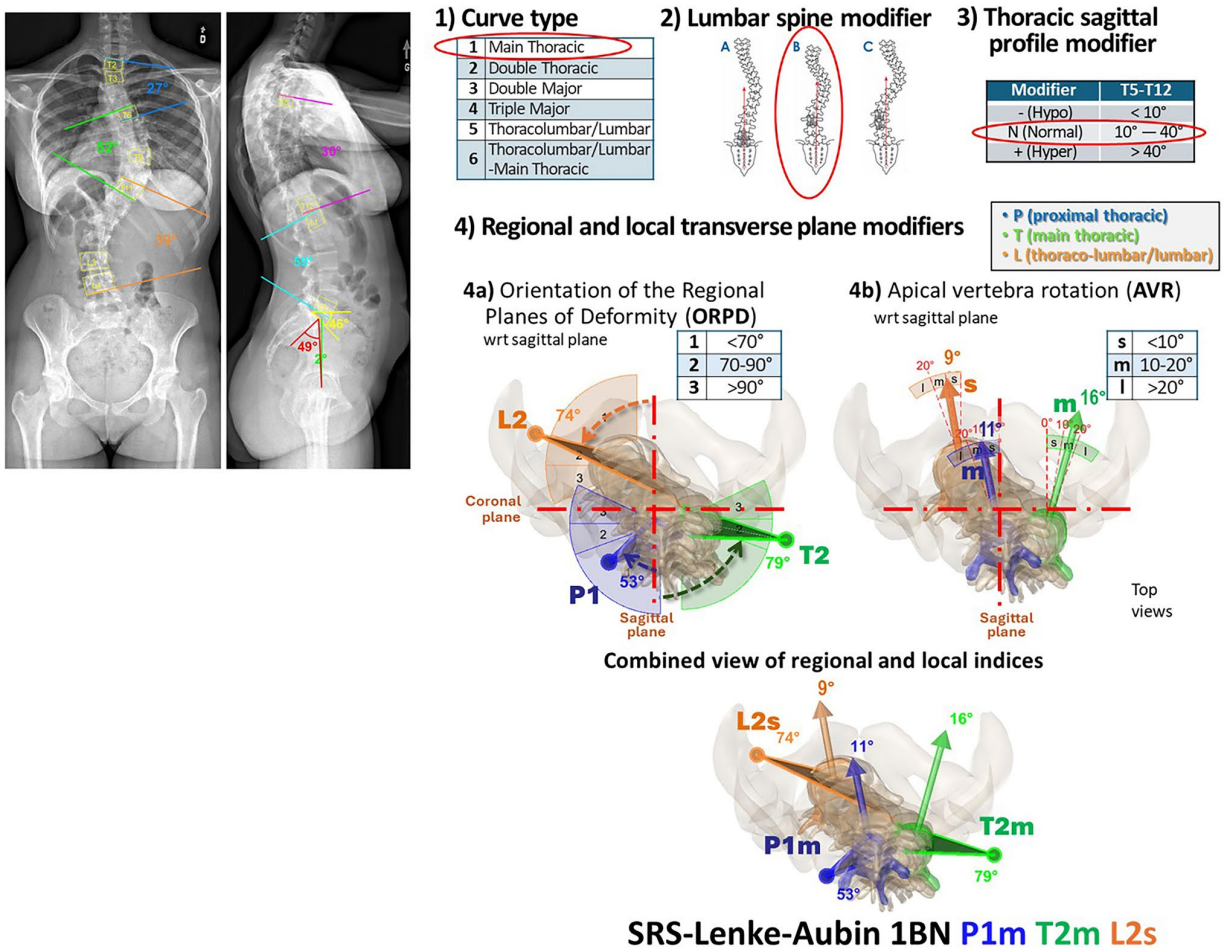
Case example illustrating the integration of the regional and local transverse plane modifiers into the new SRS–Lenke–Aubin 3D Classification. SRS–Lenke–Aubin 3D classification = curve type (1–6) + lumbar spine modifier (A, B, C) + thoracic sagittal modifier (–, N, +) + transverse plane modifiers for the P, T and L regions: ORPD (1, 2, 3) and AVR (s, m, l). A combined view of the regional and local transverse plane modifiers is displayed below. In this case example, the resulting new classification is SRS–Lenke–Aubin 1BN P1m T2m L2s. By convention, the PT, MT, and TL/L regions are represented respectively in blue, green, and orange

The format and application of the classification are illustrated using the example shown in Fig. 5.

### Analysis of distribution of transverse plane local and regional modifiers

The objective of this analysis was to evaluate how the transverse plane modifiers perform in real-world clinical settings. Specifically, it aimed to quantify how cases were distributed across the full 3 × 3 subclass matrix and determine whether all possible subclass combinations were represented in the cohort. This approach was intended to assess the generalizability and robustness of the proposed classification. To do so, a retrospective sub-analysis was performed on a cohort of 285 surgically treated AIS patients who underwent 3D reconstruction from biplanar radiographs (35% Lenke 1; 21% Lenke 2; 14% Lenke 3; 8% Lenke 4; 13% Lenke 5, and 9% Lenke 6). For each patient, the ORPD and the AVR were computed across the PT, MT, and TL/L segments. These measurements were systematically mapped to their corresponding regional (ORPD) and local (AVR) subclass tiers using the thresholds previously defined.

## Results

Figure 5 illustrates a detailed case example demonstrating the integration of regional and local transverse plane modifiers within the SRS–Lenke–Aubin 3D Classification. Shaded reference rulers aid in subclass identification by visually delineating category thresholds. Item 4a highlights the ORPD index,  item 4b shows the AVR index, and  bottom row presents the combined visualization of regional and local modifiers.

**Fig. 6:**
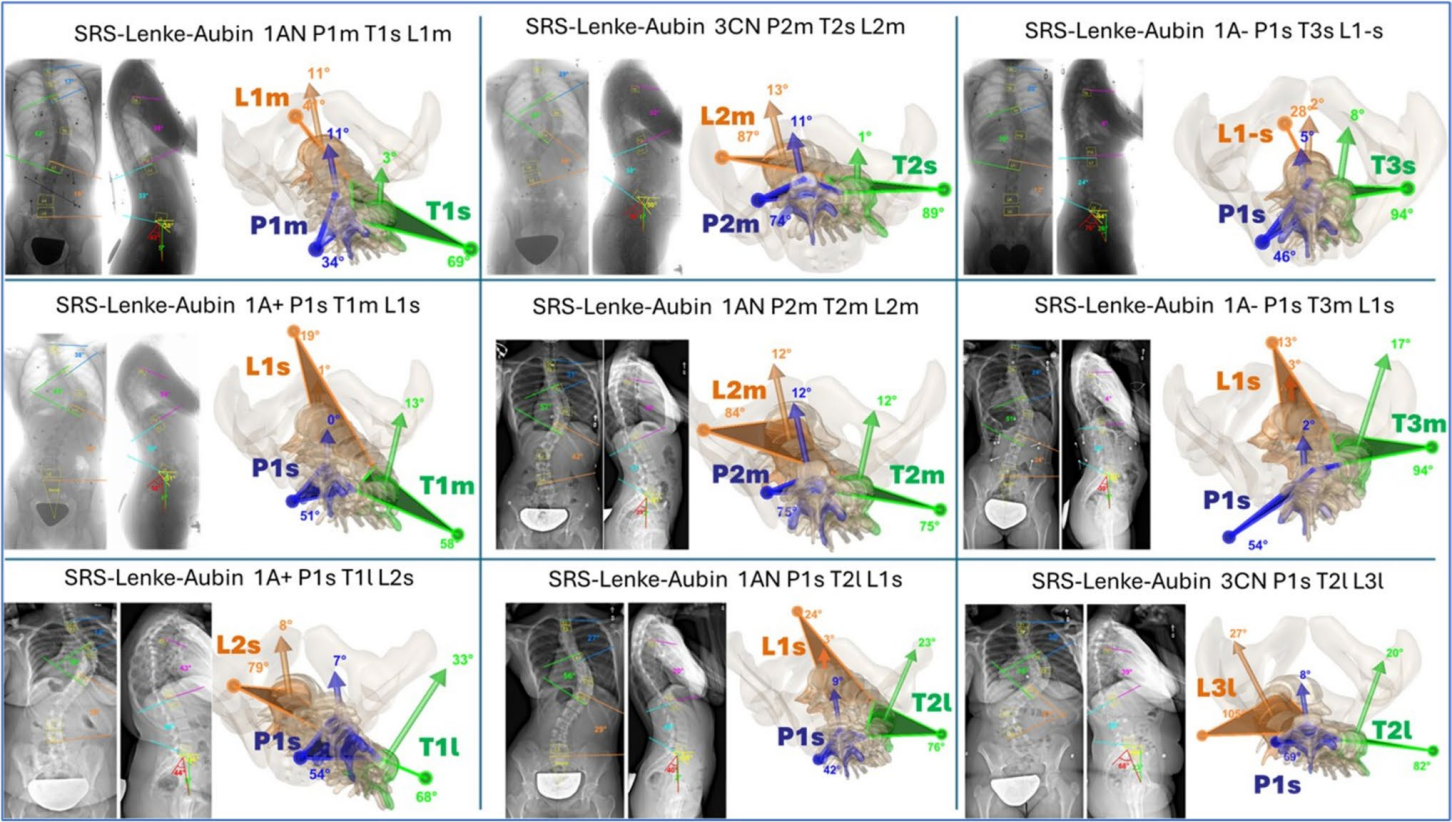
Representative examples of transverse plane indices and their corresponding classifications within the SRS–Lenke–Aubin 3D system. The first row features cases with predominantly small (s) AVR values (< 10°), the middle row shows examples with intermediate (m) AVR (10°–20°), and the bottom row illustrates cases with more pronounced (l) AVR (> 20°). The left, middle, and right columns present cases in which ORPD types 1, 2, and 3 are respectively predominant, although mixed patterns may still be observed within each group

Figure 6 presents additional representative examples of transverse plane indices and their corresponding classifications within the SRS–Lenke–Aubin 3D system. These cases, drawn from a spectrum of AIS curve patterns, illustrate how regional (ORPD) and local (AVR) measurements—obtained from 3D reconstruction data—are used to assign transverse plane modifiers to each spinal segment (PT, MT, and TL/L). The first row features cases with predominantly small (s) AVR values (< 10°), the middle row shows examples with intermediate (m) AVR (10°–20°), and the bottom row illustrates cases with more pronounced (l) AVR (> 20°). The left, middle, and right columns group cases in which ORPD types 1, 2, and 3 are respectively predominant, while acknowledging that mixed or transitional features may still be present within each column and row. These indices provide a concise yet clinically meaningful characterization of spinal deformities and serve as the foundation for segment-level classification within the integrated framework.

**Fig. 7:**
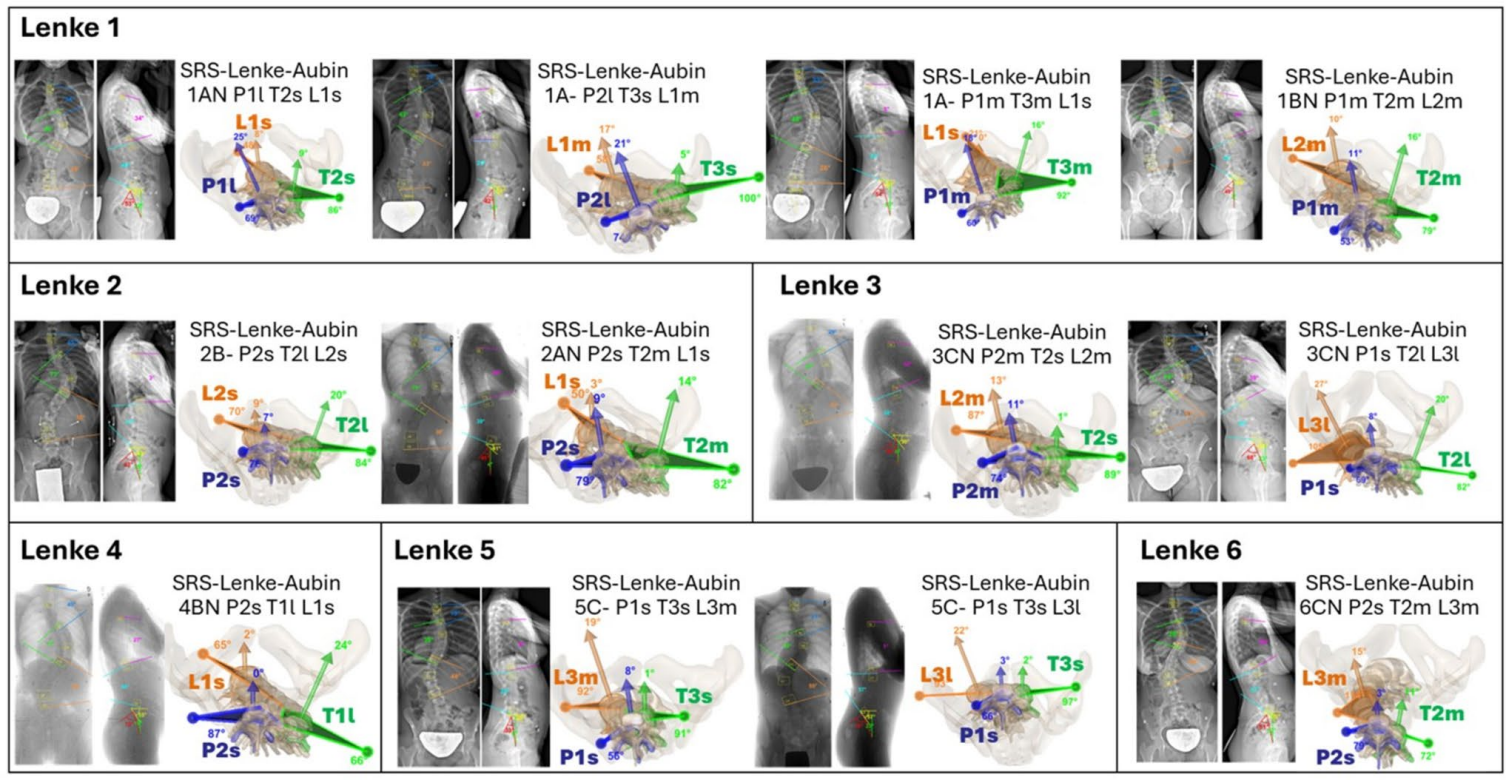
Representative cases from various Lenke categories, illustrating the range of 3D morphological variations captured by the SRS–Lenke–Aubin 3D classification. These examples highlight structural differences that are not readily apparent in conventional 2D analysis and complement the standard Lenke classification by providing additional transverse plane information

Figure 7 further illustrates this approach by presenting representative cases from different Lenke categories, showcasing the diversity of 3D morphological patterns that can occur within similar 2D classifications. These examples emphasize how the 3D classification complements the conventional Lenke system by revealing transverse plane features that are not readily captured by standard 2D analysis.

**Fig. 8:**
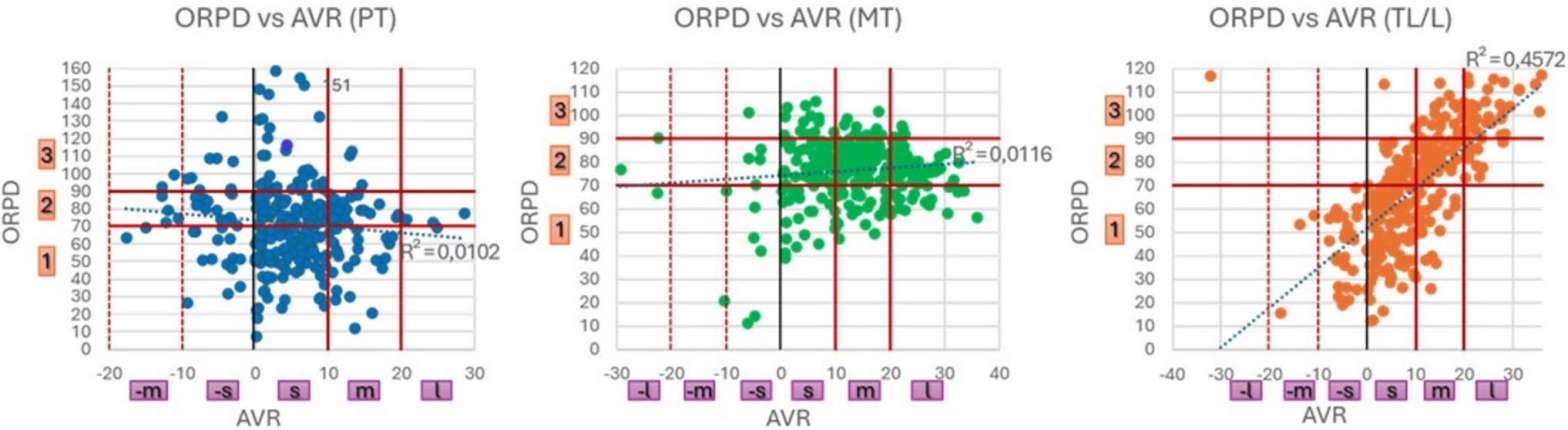
Relationship between ORPD and AVR for both the PT (left), MT (center) and TL/L (right) segments. The scatter plots illustrate the diversity of transverse plane deformities observed in the cohort, with each point representing a case and data points distributed across all quadrants formed by the combination of three regional deviation classes (ORPD 1–3) and three apical vertebral rotation classes (s, m, l), including cases with negative (inverse) AVR

Expanding beyond these illustrative examples, Fig. 8 presents the distribution of transverse plane indices across the entire 285-case surgical cohort. The combined ORPD and AVR values revealed a wide spread across the 3 × 3 matrix, confirming the system’s capacity to capture the diversity of AIS deformities without prior stratification by Lenke type. All nine subclass combinations—from 1s to 3l—were collectively represented across the PT, MT, and TL/L segments, though not all were present within each segment individually. This highlights the overall discriminative power and robustness of the classification framework in capturing the diversity of AIS deformities. ORPD values ranged from below 70° (ORPD 1) to above 90° (ORPD 3), reflecting varying degrees of regional deviation, including cases of sagittal inversion. Concurrently, AVR values ranged from minimal (< 10°) to pronounced (> 20°) apical rotation, as well as negative values, illustrating the heterogeneity of local vertebral torsion across patients.

Importantly, both Figs. 6, 7 and 8 emphasize that curves with similar coronal profiles can exhibit markedly different transverse plane characteristics. Moreover, for a given ORPD value, a wide range of AVR values was observed—highlighting the partial independence between these two descriptors. This observation is quantitatively supported by low coefficients of determination (r2) between ORPD and AVR: below 0.1 for the PT and MT segments, and moderate (r2 = 0.4572) for the TL/L segment. These results reinforce the necessity of including both regional and local transverse plane indices to adequately capture the three-dimensional complexity of AIS.

## Discussion

The SRS–Lenke–Aubin 3D classification system presents a pragmatic, clinical approach to integrate three-dimensional descriptors into AIS evaluation. While scoliosis is fundamentally a complex 3D deformity, this system purposely distills its spatial intricacies into a concise set of two distinct segment-level indices—ORPD and AVR—that are intuitive, reproducible, and communicable. Though the classification uses categorical descriptors, it is anchored in continuous measurement, ensuring interpretability without oversimplification. This classification is intended to support standardization across practitioners and facilitate communication and decision-making. The selected thresholds (70° and 90° for ORPD; 10° and 20° for AVR) effectively stratify clinically meaningful subtypes: ORPD > 90° was consistently associated with sagittal curvature inversion, 70–90° with sagittal flattening, and AVR > 20° with significant vertebral torsion—confirming the clinical relevance of these thresholds.

Importantly, the classification applies thresholds to inherently continuous variables, acknowledging that spinal deformity occurs along a continuum rather than in discrete clusters. As such, cases near threshold boundaries (Figs. 4, 8) may require nuanced interpretation, since small numerical shifts may influence subclass assignment without reflecting abrupt clinical changes. These borderline cases emphasize the need to interpret the 3D indices within a broader clinical context. The segmental structure of the descriptors (PT, MT, TL/L), aligned with the original Lenke framework, facilitates such integrative reasoning by situating transverse plane metrics within the full 3D architecture of the spine. Given its segment-based architecture, the system may naturally lend itself to flexible use, whereby clinicians could choose to emphasize only the modifiers most relevant to a given case—much as occurred with the original Lenke system—thereby reducing cognitive load while retaining the option to incorporate additional descriptors in more complex or ambiguous situations.

Examples shown on Figs. 6 and 7 highlight that curves with similar coronal profiles can exhibit markedly different transverse plane characteristics—such as increased regional deviation or pronounced apical rotation—underscoring the added value of explicitly incorporating 3D descriptors. This further illustrates the system’s ability to capture the complex 3D architectural reorganization of the spine that characterizes AIS, and the classification’s ability to distinguish clinically relevant patterns of transverse plane deformity at the segmental level. By integrating both regional and local descriptors, the SRS–Lenke–Aubin 3D system offers a more anatomically faithful and comprehensive representation of AIS deformities, ultimately supporting more personalized and targeted surgical planning. Examples shown on Figs. 6 and 7 highlight that curves with similar coronal profiles can exhibit markedly different transverse plane characteristics—such as increased regional deviation or pronounced apical rotation—underscoring the added value of explicitly incorporating 3D descriptors. This further illustrates the system’s ability to capture the complex 3D architectural reorganization of the spine that characterizes AIS, and the classification’s ability to distinguish clinically relevant patterns of transverse plane deformity at the segmental level. By integrating both regional and local descriptors, the SRS–Lenke–Aubin 3D system offers a more anatomically faithful and comprehensive representation of AIS deformities, ultimately supporting more personalized and targeted surgical planning. While larger curves may exhibit more pronounced 3D characteristics, our data also show substantial heterogeneity in ORPD and AVR values among curves of similar Cobb magnitude, indicating that these transverse plane descriptors capture clinically relevant morphology beyond curve size alone. At the same time, we acknowledge that the system introduces a level of complexity that reflects the true multidimensional nature of spinal deformity; this complexity is not incidental but represents a deliberate and worthwhile investment to capture clinically meaningful 3D information that cannot be conveyed in simpler frameworks.

This original structured yet flexible design distinguishes the classification from earlier 3D systems that relied on abstract or computationally complex constructs, such as fuzzy clustering, geometric torsion, da Vinci projections, or planes of maximal curvature [23, 24, 26, 28]. While conceptually rich, those approaches often faced limited clinical uptake due to interpretability and workflow constraints.

By contrast, the new SRS–Lenke–Aubin system builds upon the familiar modular logic of the original Lenke 2D modular classification. Its extension through ORPD and AVR modifiers enables clinicians to analyze transverse plane deformities without abandoning established diagnostic routines. This continuity makes the system particularly suitable for integration into surgical planning. Each of the regional ORPD informs curvilinear correction trajectories that address both coronal and sagittal components, defining an arc-like repositioning path rather than relying solely on uniplanar translation. Simultaneously, AVR offers insight into localized vertebral torsion, guiding decisions about targeted derotation strategies—such as direct vertebral rotation or instrumentation adjustment—crucial for achieving true 3D correction.

Notably, both ORPD and AVR are intuitive metrics: they represent deviations from the sagittal plane and are typically corrected toward 0° during surgery. This target value reflects the anatomical norm but can also be adjusted to a value that optimizes global spinal balance in all three planes. The ideal correction is therefore not necessarily absolute neutralization, but rather a biomechanically sound alignment that harmonizes the regional correction within a holistic 3D strategy. Used in tandem, these indices holistically support a more individualized and biomechanically coherent surgical plan, tailored to the patient’s 3D specific regional and segmental architecture.

This balance between dimensional completeness and clinical usability is central to the system’s strength. While the lumbar modifier in the original Lenke framework primarily reflects coronal plane lumbar deviation and imbalance, it lacks integration of sagittal and transverse dimensions, ORPD introduces a more integrated spatial descriptor that incorporates sagittal and transverse context—especially critical in thoracic and thoraco-lumbar/lumbar segments. These distinctions enhance the diagnostic and planning value of the system.

Furthermore, the inclusion of ORPD, AVR, and potentially DAEVL alongside conventional radiographic parameters (Cobb angle, kyphosis, lordosis, pelvic incidence) offers a more comprehensive representation of the deformity. This broader parameter set can guide more refined, region-specific surgical objectives, such as restoring sagittal profiles and correcting torsional elements through direct maneuvers.

Compatibility with standard imaging protocols and reconstruction technologies supports the potential for broad clinical adoption. Nonetheless, we acknowledge that, as with prior 3D classification efforts, translating this system into routine clinical use will require overcoming persistent challenges related to access, workflow integration, and clinician training. In parallel, the SRS 3D Classification Task Force is actively developing an implementation plan to enable near-term deployment of the system and provide practical support for its use by SRS members and affiliated clinicians. Importantly, the classification was deliberately designed with progressive automation in mind, and ongoing SRS initiatives specifically aim to enable its application without requiring clinicians to manually perform 3D reconstructions. While these initiatives aim to mitigate previously reported barriers [7, 31], further work will be needed to evaluate their real-world impact and ensure sustainable integration into clinical practice.

Beyond the current transverse plane metrics, integrating additional descriptors such as pelvic parameters, thoracic cage morphology, shoulder asymmetry, or global balance alignment could further enrich the classification. As it stands, this work represents the foundational level of a broader 3D-informed classification system—an initial structural tier designed to evolve. The potential to expand this framework with complementary indices underscores its scalability and long-term relevance across the spectrum of scoliosis management, from diagnosis to surgical planning and follow-up.

Looking ahead, multi-center validation studies will be essential to confirm reliability and assess clinical impact. In parallel, the new transverse plane descriptors introduced by the SRS–Lenke–Aubin 3D classification may support improved 3D correction strategies. These include region-specific adjustments in rod contouring, targeted direct vertebral derotation, and arc-based correction trajectories informed by regional plane orientation—each tailored to the patient’s 3D spinal architecture. The complementary value and relative independence of these transverse plane descriptors—together with illustrative examples of curves showing similar coronal patterns but distinct 3D characteristics—are explored in greater detail in a companion manuscript (Aubin et al. 2025), which highlights how transverse plane analysis can reveal morphological distinctions not apparent on standard radiographs. Future developments may include integration with automated 3D analysis platforms, enabling real-time classification from routine radiographs. An important next step will also be to determine the clinical scenarios in which these 3D descriptors offer added value for surgical planning beyond conventional 2D assessment. By facilitating the incorporation of 3D measurements into standard workflows, the SRS–Lenke–Aubin 3D system provides a concrete step toward more individualized and complete geometry-driven care in AIS. Beyond its surgical applications, the system’s regional and multiplanar approach may also prove valuable for refining bracing strategies in non-operative treatment, for improving risk stratification based on 3D curve profiles, and for potential adaptation to adult scoliosis assessment, where complex 3D deformities are also encountered.

## Data Availability

In the spirit of open science, inquiries regarding potential use of the data for future research may be directed to the corresponding author. Access is subject to ongoing studies of the SRS 3D Classification Task Force currently making use of these datasets.
