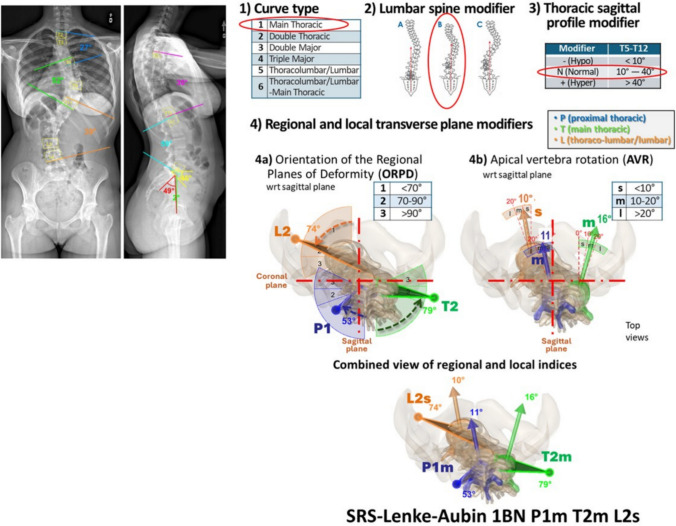# Correction: The SRS–Lenke–Aubin 3D classification of adolescent idiopathic scoliosis

**DOI:** 10.1007/s43390-026-01393-z

**Published:** 2026-05-23

**Authors:** Carl-Eric Aubin, Lawrence G. Lenke, Michael Vitale, Justin S. Smith, Virginie Lafage, Michelle C. Welborn, A. Noelle Larson, Takashi Kaito, Peter O. Newton, Jeffrey Mullin, Christiane Caouette, Brice Ilharreborde

**Affiliations:** 1 Department of Mechanical Engineering, Polytechnique Montréal, Downtown Station, P.O. Box 6079, Montreal, QC H3C 3A7 Canada; 2 Sainte-Justine University Hospital Center, 3175 Côte Sainte-Catherine Road, Montreal, QC H3T 1C5 Canada; 3 Department of Orthopedic Surgery, Columbia University Vagelos College of Physicians and Surgeons, NewYork-Presbyterian Och Spine Hospital, New York, NY USA; 4 Department of Orthopedic Surgery, Columbia University Vagelos College of Physicians and Surgeons, NewYork-Presbyterian Morgan Stanley Children’s Hospital, New York, NY USA; 5 Department of Neurosurgery, University of Virginia School of Medicine, Charlottesville, VA USA; 6 Department of Orthopaedic Surgery, Lenox Hill Hospital, Northwell Health, New York, NY USA; 7 Department of Orthopaedics and Rehabilitation, Oregon Health and Science University School of Medicine, Shriners Children’s Portland, Portland, OR USA; 8 Department of Orthopedic Surgery, Mayo Clinic, 200 First Street SW, Rochester, MN 55905 USA; 9 Division of Orthopaedic Surgery, Osaka University Graduate School of Medicine, Osaka Rosai Hospital, Osaka, Japan; 10 Department of Orthopedics, Rady Children’s Hospital, 3020 Children’s Way, San Diego, CA 92123 USA; 11 Department of Neurosurgery, Jacobs School of Medicine and Biomedical Sciences, University at Buffalo, The State University of New York, Buffalo, NY USA; 12 AP-HP Hôpital Universitaire Robert-Debré, 48 Boulevard Sérurier, 75019 Paris, France

**Correction to: Spine Deformity** 10.1007/s43390-025-01253-2

Figure 5 as originally published was incorrect (the lumbar AVR was inadequately rounded). The correct figure is

**Fig. 5:**
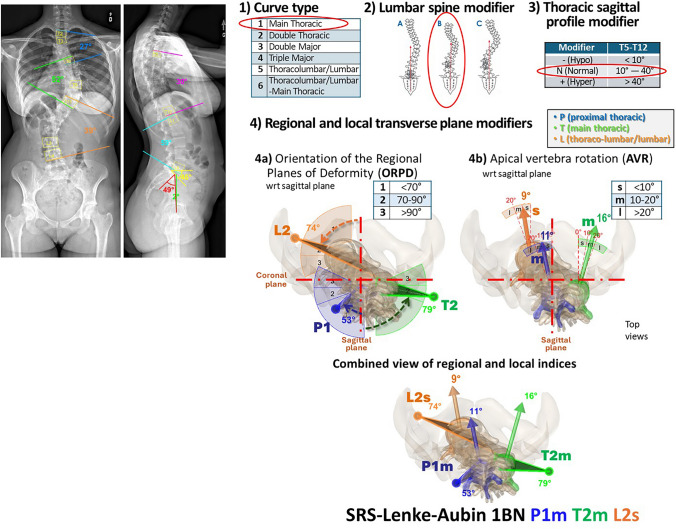
Case example illustrating the integration of the regional and local transverse plane modifiers into the new SRS–Lenke–Aubin 3D Classification. SRS–Lenke–Aubin 3D classification = curve type (1–6) + lumbar spine modifier (A, B, C) + thoracic sagittal modifier (–, N, +) + transverse plane modifiers for the P, T and L regions: ORPD (1, 2, 3) and AVR (s, m, l). A combined view of the regional and local transverse plane modifiers is displayed below. In this case example, the resulting new classification is SRS–Lenke–Aubin 1BN P1m T2m L2s. By convention, the PT, MT, and TL/L regions are represented respectively in blue, green, and orange

The original article has been updated.

The incorrect version is included below for transparency.